# The Effect of the Embodied Guidance in the Insight Problem Solving: An Eye Movement Study

**DOI:** 10.3389/fpsyg.2018.02257

**Published:** 2018-11-26

**Authors:** Qiang Xing, Cuiliang Rong, Zheyi Lu, Yanfeng Yao, Zhonglu Zhang, Xue Zhao

**Affiliations:** ^1^Department of Psychology, School of Education, Guangzhou University, Guangzhou, China; ^2^Jiangcun Primary School, Guangzhou, China; ^3^Shunde Experiment Middle School, Foshan, China

**Keywords:** insight problem solving, creativity, attention guidance, embodied effect, eye movement track

## Abstract

Insight is an important cognitive process in creative thinking. The present research applied embodied cognitive perspective to explore the effect of embodied guidance on insight problem solving and its underlying mechanisms by two experiments. Experiment 1 used the matchstick arithmetic problem to explore the role of embodied gestures guidance in problem solving. The results showed that the embodied gestures facilitate the participants’ performance. Experiment 2 investigated how embodied attention guidance affects insight problem solving. The results showed that participants performed better in prototypical guidance condition. Experiment 2a adopted the Duncker’s radiation problem to explore how embodied behavior and prototypical guidance influence problem solving by attention tracing techniques. Experiment 2b aimed to further examine whether implicit attention transfer was the real cause which resulted in participants over-performing in prototypical guidance condition in Experiment 2a. The results demonstrated that overt physical motion was unnecessary for individuals to experience the benefits of embodied guidance in problem solving, which supported the reciprocal relation hypothesis of saccades and attention. In addition, the questionnaire completed after experiments showed that participants did not realize the relation between guidance and insight problem solving. Taken together, the current study provided further evidence for that embodied gesture and embodied attention both facilitated the insight problem solving and the facilitation is implicit.

## Introduction

Insight is an important cognitive process in creative thinking. Exploring insight and its underlying mechanism helped us understand creative thinking better. Insight is a special form of problem solving, namely insight problem solving. Other than solving general problem, individuals cannot specifically explain problem solving steps or process; insight problem solving is an “aha” experience in which participants suddenly and intuitively understand complex situations or seize the key to the problem (Bowden and Jung-Beeman, 2007). The well-established cognitive theories interpreting insight problem solving are representational change theory, progress monitor theory, and prototype heuristic theory (Zhang et al., 2004). However, recently increasing attention has been focused on how individual’s body (*e.g., feelings*, *motion*, and *active state*) influences problem representation and transformation (Stepper and Strack, 1993; Williams and Bargh, 2008; Ball and Litchfield, 2017). It has been realized that human cognition relies on body and sensory motor system, while body plays an essential role in the cognitive process. The bodied behavior is not only influenced by internal cognitive processes, but also affects cognitive processes conversely (Brouillet et al., 2010; Hao-Sheng, 2011; Jones, 2017). How do embodied behavior influence insight problem solving, and could embodied cognition theory interpret insight problem solving process? Although some studies have found that embodied behavior and active state promotes problem solving (Bowden and Jung-Beeman, 2007; Cook et al., 2008; Cook and Tanenhaus, 2009; Schubert and Semin, 2009; Werner and Raab, 2014), how the effect works is not clear. Hence, it is necessary to explore how embodied behavior and active state influences insight problem solving using embodied cognition theory. The present study aimed to explore the function and underlying mechanisms about how embodied guidance affect insight problem solving.

Embodied cognitive can be understood that cognition is influenced by the environment and the body, including its potential actions (Adam and Galinsky, 2012; Goldinger et al., 2016). Embodied effect refers to changes in cognition, attitude, social perception, emotion, and others related to the tasks involved when experience or simulate the movement or state of body, and this kind of functional dependence theory is embodied theory (Qiu-Ping et al., 2011; Horchak et al., 2014). According to embodied theory, bodied behavior and states of the body could change cognitive status (Wilson and Sabrina, 2013). The insight experience is a special experience in the process of insight problem solving, and it existed in the whole process of insight problem solving (Shen et al., 2015). Shen et al. (2018b) showed that insight experience was a complex, multidimensional construction with cognitive, affective, and embodied characteristics. Some researches supported that insight problem solving or insight experience is embodied (Leung et al., 2012; Jarman, 2014). And some studies found that gestures or speech could help to understand knowledge and solve problems. The information of problem would lead into mental representation by gestures or speech, then promote thinking and problem solving (Broaders et al., 2007; Cook et al., 2008; Cook and Tanenhaus, 2009; Beilock and Goldin-Meadow, 2010; Cook et al., 2010; Chu and Kita, 2011). Chu and Kita (2011) used mental rotation task and origami task to investigate whether the gesture can improve visual space problem solving, and the results showed that the group allowed to use gestures performed better in task than that not allowed to use gestures, which indicates that gestures not only reflect the process of thinking, but also affect it and then promote problem solving.

Previous research have found that embody gestures or speech play an important role in promoting insight problems solving, and others have found that body movement also affects the insight problems solving, such as patterns of eye movement or attention (Knoblich et al., 2001; Grant and Spivey, 2003; Thomas and Lleras, 2007, 2009; Litchfield and Ball, 2011; Werner and Raab, 2014). Knoblich et al. (2001) used matchstick equation problems by eye movement technology to investigate mechanism of insight problem solving. They found that the behavior of eyes gazing on problem characteristics revealed the mechanism of the predicament and insight. It was found that individuals tended to focus their look differently at the former and later stage of problem solving. Problem solving winners tended to shift their attention to the key areas before the occurrence of insight. Grant and Spivey (2003) demonstrated that there is relation between eye movement and the cognitive process. They used tumors-laser radiation problem (Duncker and Lees, 1945) and recorded eye movement of participants. The result showed that problem solving winners gazed at the skin area more and made more fixation and switch of skin-crossing in-and out. This pattern of eye movements could draw the outline of solution to problems. Litchfield and Ball (2011) found that other people’s eye movement patterns could also guide participants in insight problems solving. It is concluded that eye movement leads the cognitive process of problem solving. Based on this, can it be possible to improve the accuracy of problem solving by giving problem solvers a hint of attention or guiding them to pay attention to the key areas of problem solving? Therefore, a task was designed to guide the individuals’ eye movement and reflect the pattern of problem solving by moving their eyes, so as to explore prototypical guidance mechanism of insight solution. Research found that heuristic prototype was important in solving scientific innovation insight problems (Yang et al., 2016).

In addition, it was still controversial whether an individual can realize the process of insight problem solving. Most research found that the process of insight problem solving is implicit, and individuals do not realize the hints of problem solving (Grant and Spivey, 2003; Ollinger et al., 2013; Riffert, 2013; Branchini et al., 2016). Ollinger et al. (2013) on eight-coin problem found that implicit use the third dimension to find the solution. Conversely, some research found individuals are aware of the hints or trains (Dow and Mayer, 2004; Patrick and Ahmed, 2014). It may be related to feature of the problem hints or trains. Therefore, we intended to explore whether individuals realize the connection between embodied guidance and problem solving in this study, and it is inferred that the process of insight problem solving is implicit or explicit.

To sum up, some studies have found that embodied behavior and eye movement could guide individual thinking, thus affect problem solving. However, it is still not clear what the underlying mechanism is between the embodied active state and cognition. Therefore, we used the eye movement technology to explore how embodied guidance influences insight problem solving and its underlying mechanisms. And whether the embodied effect of insight problem solving truly need external behavior or just only internal attention-transfer? The present study consisted of two experiments, respectively, to explore the effects of embodied behavior and embodied attention guidance on insight problem solving, and the mechanism of embodied effect in insight problem solving. In Experiment 1, we used matchstick arithmetic problem to explore the role of embodied behavior (*gestures guidance* and *speech guidance*) in problem solving. The matchstick arithmetic problem is the most suitable experimental material to explore the problem representation influencing the problem solving for it has different degrees of representation transformation. In Experiment 2a, we adopted the Duncker’s radiation problem to explore how embodied behavior and prototypical guidance influence problem solving by attention tracing techniques. In Experiment 2b, aimed to further examine whether implicit attention transfer was the real cause which resulted in participants over-performing in prototypical guidance condition in Experiment 2a. We used Duncker’s radiation problem as Experiment 2 material because the components of the problem and its answer are relatively simple. There are three components (*tumor*, *healthy tissue*, and *skin outside*) and the answer involves only two key components (*low-density multiple lasers*). It not only allows us to achieve embodied attention guidance through digital tracking tasks, but also to operate different digital tracking tasks to study the impact of prototype heuristic on the insight problem solving.

## Experiment 1

### Materials and Methods

#### Participants

Ninety-two undergraduate students (55 females, mean age = 22.56 years, *SD* = 3.82) were recruited for course credit or proper reward. All participants reported normal or corrected to normal vision. They signed the informed consent and they had not participated in similar experiments before. All participants were randomly assigned to four groups.

#### Design

The design of Experiment 1 was a single factor between participants. The independent variable is guidance pattern (gestures guidance, speech guidance, mix guidance, no guidance). The accuracy, reaction time, fixation duration, and the number of fixation of each areas of interest (AOI) are dependent variable.

#### Materials and Apparatus

We collected 70 matchstick arithmetic problems in total by looking up the online math problem library and various books. All arithmetic did not exceed two digits (41 addition; 29 subtraction), including 40 numerical constraint problem, 30 symbol constraint problem. Numerical constraint problem refers to that participants need to change the figure to make equation valid in this kind of problem while they have to change the operation symbol in symbol constraint problem. All materials were made in Photoshop with font being Time New Roman, font size being 72, white background, and black character.

**FIGURE 1:**
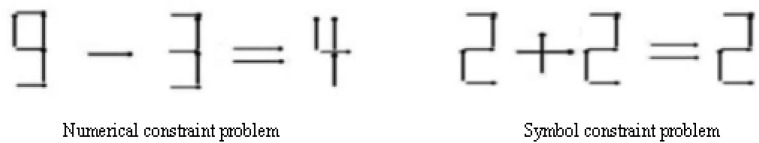
Matchstick arithmetic problems type diagram.

Sixteen additional undergraduate students (7 females, mean age = 20 years, *SD* = 2), who had no prior experience in solving matchstick arithmetic problems, were asked to evaluate the difficulty of problems by five points. In order to balance the order of problems, half of the participants made evaluation in the order from the front to the back, while the other half otherwise. Finally, 23 matchstick arithmetic problems (excluding 17 more than the only answer problems, 11 interacted problems, and 19 too hard or too easy problems) were chosen as experiment materials. The difficulty is moderate (*M* = 2.87). The numerical constraint problem is more difficult than symbolic constraint problem. The problem type of matchstick arithmetic is shown in Figure 1.

An SR Research (Mississauga, ON, Canada) EyeLink Plus Eye-Tracking System recorded participants’ eye movements with a sampling rate of 500 Hz. This eye tracker has a high spatial (0.01° of visual angle) using pupil tracking and corneal reflection. The materials were displayed on a 19-in Dell monitor with a refresh rate of 75 Hz (resolution 1,024 × 768 pixels), and the viewing distance was approximately 60 cm. Viewing was binocular and only right eye was tracked as permitted by the quality of the calibration for right eye. The experiment was run with E-Prime 1.10 software.

#### Procedure

There were four experimental conditions: Gesture guidance condition (G): after each problem is presented, participants were asked to draw a horizontal line on the left of it using left hand and another one using right hand. Speech guidance condition (S): after each problem is presented, participants were asked to utter the phase “How can you do to complete the equation by moving only one matchstick?” Mix guidance (M): after each problem is presented, participants were asked to perform both. Control (no guidance) condition (C): without any guidance.

There were 23 trials in Experiment 1. The matchstick arithmetic problems were lasting on the screen for 50 s, during which participants were asked to make the equation valid by moving only one matchstick. If participants came up with the answer, they should report it orally and the experimenter would record it, otherwise the next problem would be presented after 50 s.

**FIGURE 2:**
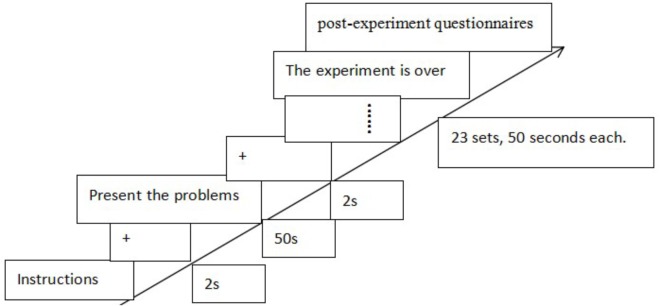
The detailed procedures of Experiment 1.

At the end of the experiment, participants were asked to assess the difficulty of the problem by five point, whose scale ranging from 1 (*very difficult to solve*) to 5 (*very easy to solve*). The sense of surprise about problems solving by five points, whose scale ranging from 1 (*very surprised*) to 5 (*not surprised at all*). Participants should also answer that whether they realize the connection between guidance and problem solving? 1 refers to no relation, 2 guidance providing clues and hints, and 3 guidance interfering thinking. The detailed procedures of the experiment are in Figure 2.

### Results

#### Post-experiment Questionnaires Analyses

Post-experiment questionnaires aimed to find out whether participants realized the connection between guidance and problem solving, and the results showed that 5 participants realized that there was a link between the guidance and problem solving (2 in G condition, 3 in M condition), the other 87 participants didn’t realize the connection. Data of 85 participants were in final analysis excluding 5 realizing the connection and 2 uncompleted recording. The results of difficulty level and the sense of surprise about problem solving are shown in Table 1.

**Table 1:** Evaluation of difficulty level and the sense of surprise about problem solving (one).

Guidance condition	Difficulty level of problem					A sense of surprise				
	1	2	3	4	5	1	2	3	4	5
G	1	5	13	3	0	0	12	7	3	0
S	2	9	9	1	0	2	8	9	1	1
M	0	9	6	5	0	0	8	4	8	0
C	0	9	12	1	0	0	7	12	1	2

*G is the gesture guidance condition; S is the speech guidance condition; M is the mix guidance condition; and C is the control condition.*

Chi-square test showed that there was no significant difference in four experimental conditions: χ_(9)_^2^ = 12.634, *p* = 0.180. It indicated that experimental condition did not affect the solution of the problem. There was a significant difference in the sense of surprise: χ_(12)_^2^ = 25.953, *p* = 0.011, suggesting that participants had different “aha” experience in the four experimental conditions.

#### Accuracy, Reaction Time, and Ratio of Accuracy Analyses

**FIGURE 3:**
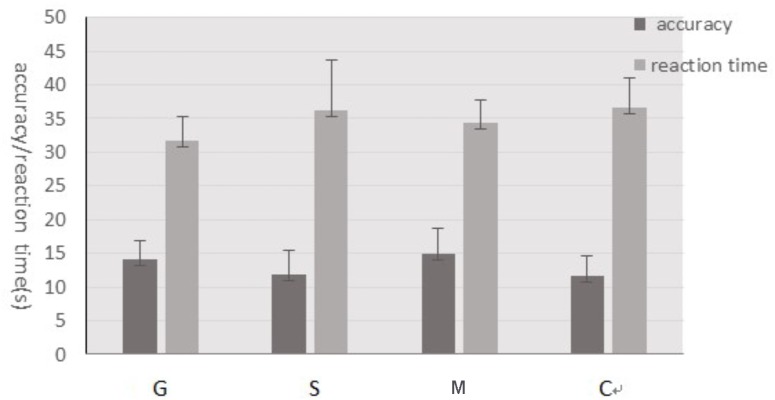
Accuracy and reaction time of the participants under different guidance conditions (error bars: 95% confidence interval).

To test the effect of different guidance conditions on insight problem solving, we analyzed the accuracy and response time in different guidance conditions. And we analyzed the ratio of accuracy in different guidance conditions to test the effect of different guidance conditions on problem type. A one-way ANOVA showed that there was a significant difference in accuracy under different experimental conditions, *F*_(3,81)_ = 4.863, *p* = 0.004, $\eta_{p}^{2}$ = 0.152. And there was a significant difference in reaction time under different experimental conditions, *F*_(3,81)_ = 4.208, *p* = 0.008, $\eta_{p}^{2}$ = 1.348. After the Tukey-HSD examination, accuracy in both G and M condition was significantly higher than that in S condition (*p* = 0.043, *p* = 0.004). Accuracy in both G and M condition was significantly higher than C condition (*p* = 0.023, *p* = 0.002). Response time in G condition was significantly shorter than C condition (*p* = 0.002). There were not significant different in other conditions (*p* > 0.05). The result showed that the accuracy was the highest and the response time was shortest in the gesture guidance condition. It suggested that the gesture guidance promoted the insight problem solving. The results were shown in Figure 3.

**FIGURE 4:**
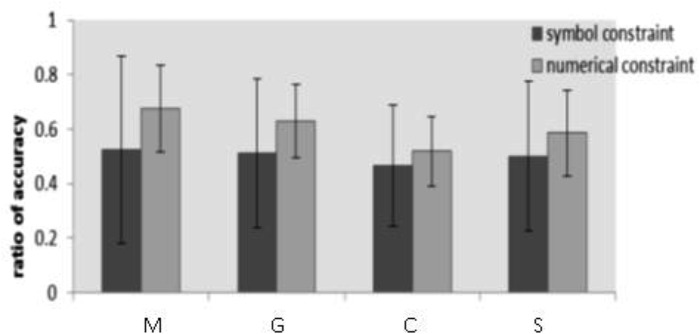
Ratio of accuracy under different guidance condition (error bars: 95% confidence interval).

A two-way repeated measures ANVOA with guidance condition and problem type on ratio of accuracy showed that the main effect of guidance condition was not significant, *F*_(3,81)_ = 1.590, *p* = 0.098, $\eta_{p}^{2}$ = 0.056. The main effect of problem type was significant, *F*_(3,81)_ = 8.077, *p* = 0.006, $\eta_{p}^{2}$ = 0.091, indicating that participants solved more numerical constraint problems (*M* ±*SD* = 0.499 ± 0.03). The interaction between guidance condition and problem type was not significant (*p* > 0.05). The results were shown in Figure 4.

#### The Total Fixation Duration and Number of Fixation Analysis

**FIGURE 5:**
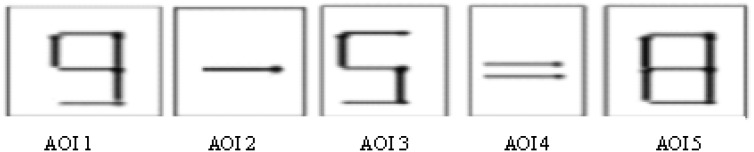
Each AOIs.

To explore the underlying mechanism of the insight problem solving, we analyzed the total fixation duration and number of fixation. First, the AOIs were defined according to the research purpose and hypothesis. In this study, each component of the matchstick arithmetic equation (figure and symbol) was defined as AOI, and the specific division of AOI is shown in Figure 5.

A three-way repeated measures ANVOA with problem type, guidance condition, and AOIs on duration of fixation showed that the main effect of the problem type was not significant, *F*_(1,81)_ = 1.516, *p* = 0.222, $\eta_{p}^{2}$ = 0.08. The main effect of guidance condition was not significant, *F*_(3,81)_ = 1.422, *p* = 0.242, $\eta_{p}^{2}$ = 0.07. The main effect of AOIs was significant, *F*_(4,324)_ = 249.490, *p* < 0.001, $\eta_{p}^{2}$= 0.755. The interaction of AOIs, problem type, and guidance condition was not significant, *F*_(12,324)_ = 1.097, *p* = 0.362, $\eta_{p}^{2}$ = 0.052. The interaction of problem type and guidance condition was not significant, *F*_(3,81)_ = 0.223, *p* = 0.88, $\eta_{p}^{2}$ = 0.071. AOIs interacted with guidance condition, *F*_(12,324)_ = 1.802, *p* = 0.047, $\eta_{p}^{2}$ = 0.063. For four conditions, fixation duration in the AOI 3 and AOI 4 was significantly different from the other three AOIs (*p* < 0.001). And fixation duration in AOI 3 was significantly longer than other AOIs, but fixation duration in AOI 4 was significantly shorter than other AOIs. Nothing else was different on M condition. Fixation duration in AOI 1 and AOI 5 was significantly longer than AOI 2 on G condition (*p* < 0.001, *p* = 0.012). Fixation duration in AOI 1 and AOI 5 was significantly longer than AOI 2 on C and S condition (*p* < 0.001). Two conditions were not significant in AOI 1–AOI 5, exception, M and G conditions were significantly shorter than C condition (*p* = 0.042, *p* = 0.018) in AOI 5. M was significant longer than G condition, G was significant shorter than S and C condition in AOI 2 (*p* = 0.007, *p* = 0.04, *p* = 0.018). The result suggested that the participants paid more attention to the numbers than symbols in the formula in all guidance conditions. And the participants in gesture guidance condition spent shorter time than other condition in AOI 5 and AOI 2. AOIs interacted with the problem type, *F*_(4,324)_ = 24.459, *p* < 0.001, $\eta_{p}^{2}$ = 0.324. Numerical constraints problem was significantly more symbol constraints problem on AOI 1 and AOI 5 (*p* = 0.021, *p* < 0.001). However, numerical constraints problem was significantly less than symbol constraints problem on AOI 3 and AOI 4 (*p* = 0.029, *p* = 0.002). The result showed that the participants paid more attention to the first number and result in numerical constraints problem, and more attention to the second number and equal mark in symbol constraints problem.

A three-way repeated measures ANVOA with problem type, guidance condition, and AOIs on the number of fixation showed that the main effect of problem type was not significant, *F*_(1,81)_ = 1.416, *p* > 0.05, $\eta_{p}^{2}$ = 0.019. The main effect of guidance condition was not significant, *F*_(3,81)_ = 0.988, *p* > 0.05, $\eta_{p}^{2}$ = 0.036. The main effect of the AOIs was significant, *F*_(4,324)_ = 282.449, *p* < 0.001, $\eta_{p}^{2}$ = 0.776. The interaction of AOIs, problem type, and guidance condition was not significant, *F*_(12,324)_ = 0.529, *p* > 0.05, $\eta_{p}^{2}$ = 0.037. The problem type did not interact with the guidance condition, *F*_(3,81)_ = 0.105, *p* > 0.05, $\eta_{p}^{2}$ = 0.038. AOIs interacted with guidance condition, *F*_(12,324)_ = 2.031, *p* = 0.021, $\eta_{p}^{2}$ = 0.069. For four conditions, AOI 3 and AOI 4 were significantly different from the other three AOIs (*p* < 0.001). And the number of fixation of AOI 3 was significantly longer than other AOIs, but the number of fixation of AOI 4 was significantly shorter than other AOIs. The number of fixation of AOI 1 was significantly longer than AOI 5 (*p* = 0.043) on M condition. The number of fixation of AOI 1 and AOI 5 was significantly longer than AOI 2 (*p* = 0.033, *p* = 0.007) on S condition. AOI 1 and AOI 5 were significantly longer than AOI 2 (*p* = 0.007, *p* < 0.001) on C condition. Four conditions were not significant in AOI 1 to AOI 4. However, M and G were significantly less than S and C condition (*p* = 0.022, *p* = 0.013, *p* = 0.016, *p* = 0.009) in the number of fixation of AOI 5. It suggested that participants paid more attention to the numbers in the formula in all guidance conditions. AOIs interacted with the problem type, *F*_(4,324)_ = 18.554, *p* < 0.001, $\eta_{p}^{2}$ = 0.091. Numerical constraints problem was significantly longer than symbol constraints problem on the number of fixation of AOI 1, AOI 2, and AOI 5 (*p* = 0.018, *p* = 0.031, *p* < 0.001). Numerical constraints problem was significantly shorter than symbol constraints problem on the number of fixation of AOI 4 (*p* = 0.002), and marginal significance in AOI 3 (*p* = 0.08). The result showed that participants paid more attention to the first number, symbol, and result in numerical constraints problem; and more attention to the second number and equal mark in symbol constraints problem.

### Discussion

The results of Experiment 1 showed that most participants did not realize the link between the guidance and problem solving based on post-experiment questionnaire analysis. The different guidance conditions have different effects on insight problem solving. Compared with those in the control condition, the participants in guidance condition reached higher accuracy and indeed shorter reaction time. Therefore, we can conclude that gesture guidance promotes the problem solving. The results are consistent with previous research (Cook et al., 2008). We interpreted that the gestures produced mental images in the participants’ mind, helping to complete the representational transformation of insight problem, which was the key to solving the problem. And gestures are indicative, which can lead participants to correspond to mental representation of the problem and the relevant position of the problem, and activate the connection between them (Wesp et al., 2001; Grant and Spivey, 2003; Morsella and Krauss, 2004). It is also possible that gestures can unload parts of the working memory. Researchers have shown that using both speech and gesture to express required less working memory than speech alone (Goldin-Meadow, 2001; Wagner et al., 2004). Based on the analysis of fixation time and the number of fixation in each AOI, the results showed that almost all participants paid more attention to the number regardless of the problem type, especially the second number. Meanwhile, they gazed less at the equal mark, indicating that participants preferred number in insight problem solving. Other researchers also found that the participants preferred to numerical in solving matches formula (Knoblich et al., 2001), they believed that participants short focused on the composition of problem meant that they understood the problem though a scan. While long gazed meant that the participants tried to solve the problem. The participants gazed on one element of the problem for a long time meant that they thought it was a key point in solving the problem.

## Experiment 2A

### Materials and Methods

#### Participants

Sixty-three undergraduate students (36 female, mean age = 23 years, *SD* = 3) were recruited for course credit or proper reward. All participants reported normal or corrected-to-normal vision. They signed the informed consent and they had not participated in similar experiments before. All participants were randomly assigned to three conditions.

#### Design

The design of Experiment 2a was a single factor between-participants design. The independent variable was eye movement guidance patterns (prototypical guidance, non-prototypical guidance, and non-guidance). The accuracy, reaction time, and the saccade counts were the dependent variables.

#### Materials

**FIGURE 6:**
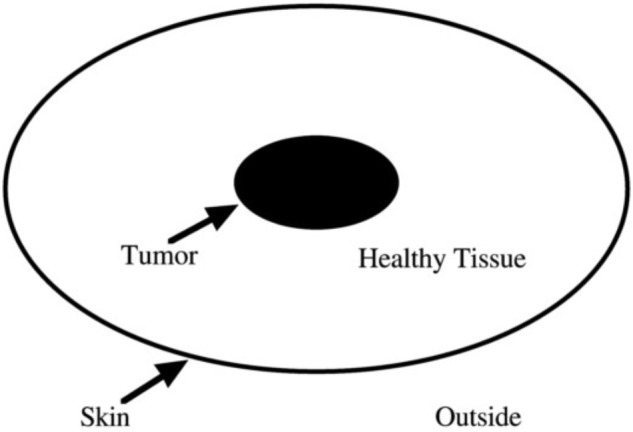
A schematic diagram of the Duncker’s radiation problem.

Radiation problem was introduced by Karl Duncker in 1945 (Figure 6). The instructions: if a person has stomach tumor and cannot be treated by surgery or medication but only by lasers radiation method. Lasers power needed to kill tumor would do harm to healthy tissue because it pass through them as well. So how can we use lasers radiation to treat patients and avoid harming other healthy tissues? There are three related areas of this problem: one is the tumor which is solid black ellipse in Figure 6. The second is the healthy tissue of the skin surrounding the tumor which is between the black ellipse and the area. The third is the area outside the skin, where the laser is emitted. The solution consists of two key components, the low-density lasers and the multiple lasers. Participants need to emit a large amount of low density and focus on the multiple lasers in the central tumor from different parts of outside the skin, only in this way can it ensure that the intensity of a single lasers won’t hurt the healthy tissue, and the multiple lasers focused on the tumor whose strength is enough to kill tumor.

**FIGURE 7:**
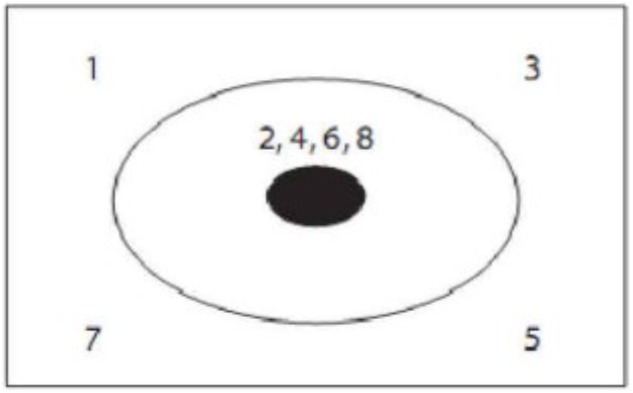
Digital tracking task of prototypical guidance condition.

**FIGURE 8:**
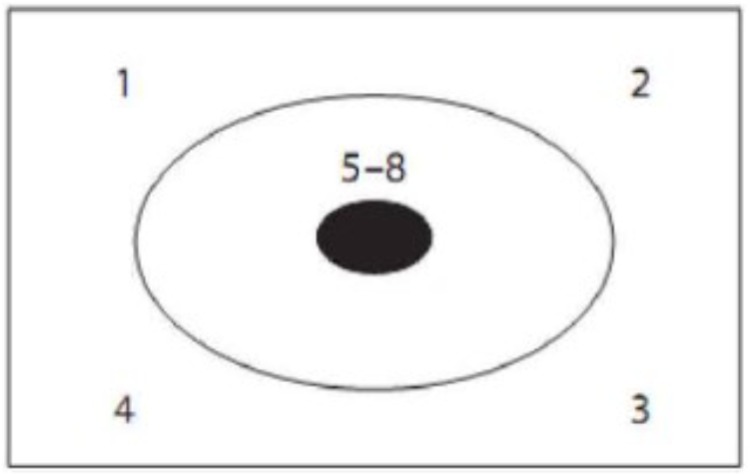
Digital tracking task of non-prototypical guidance condition.

The digital tracking task formed different guidance condition, and the digital tracking task of prototypical guidance condition is shown in Figure 7, which highlights a kind of skin-crossing or in-and-out saccades: the sight goes out of the skin into the tumor and comes out from another place. The position of numbers appearing in tracking task is: left, middle, right, middle, right, middle, left, and middle. The digital tracking task of non-prototypical guidance condition is shown in Figure 8 with different appearing position of digit: upper left, upper right, lower right, lower left, middle, middle, middle, middle. It stresses the composition of problem solving but does not highlight the key to solve the problem (emit several lasers which gather in the center of tumor from different corner). Non-guidance is designed as a control condition without digital tracking task.

#### Procedure

Experimental equipment was identical to Experiment 1. The 63 participants were randomly assigned to the experimental condition (prototypical guidance condition and non-prototypical guidance condition and non-guidance condition).

The experiment lasted for 10 min in total with 20 trails, each lasting for 30 s, including 26 s free observation time and 4 s digital tracking task. Before the start of each trail, all participants would complete a short drift correction. Then, the problem was presented and lasted for 26 s, during which participants were free to observe the problem on the screen and tried to figure out how to solve the problem. For the digital tracking task, a sequence of numbers or letters would appear on the different location of screen randomly, each lasting for 500 ms. Participants were asked to detect number and report it. The accuracy of reported number was recorded. In control condition, the participants were free to observe the problem diagram and tried to answer it without the limitation of time.

**FIGURE 9:**
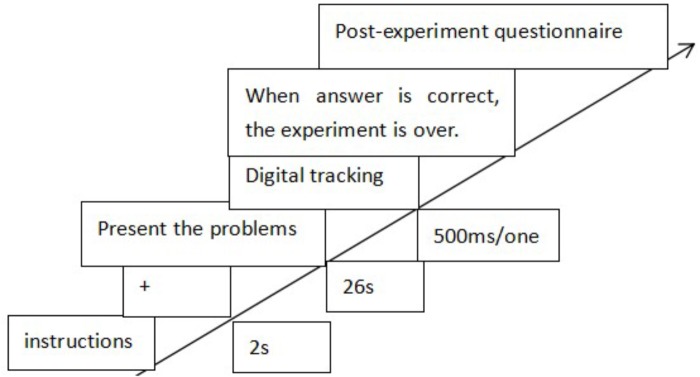
The procedures of Experiment 2a.

Once the participants came up with the answer, they could report it to the experimenter and tried to solve the problem. If the answer was correct (drawing two lines at least from different areas outside the skin pointing to the center of the tumor), the experiment was completed. If the answer was not correct, the participants would continue thinking and reporting until the end of the experiment. Participants were asked to complete a short post-experiment questionnaire, including whether participants realized the relationship between the digital tracking task and the problem, difficulty level, and a sense of surprise about problem solving, which part of the experiment (digital tracking phase, free observation phase) was the most conducive to solve the problem. The detailed procedure was shown in Figure 9.

### Results

#### Post-experiment Questionnaires Analyses

Post-experiment questionnaires intended to find out whether participants realized the relationship between the digital tracking task and the problem solving, and the results showed that three participants realized a link between the digital tracking task and problem solving. We excluded these three data in the following analysis. The specific results of difficulty level and a sense of surprise about problem solving were shown in Table 2.

**Table 2:** Evaluation of difficulty level of radiation problem and a sense of surprise about problem solving (one).

Guidance condition	Difficulty level of problem					A sense of surprise				
	1	2	3	4	5	1	2	3	4	5
Prototypical	2	8	8	2	0	1	5	7	3	4
Non-prototypical	4	3	10	2	0	2	6	7	2	2
Non-guidance	2	10	8	1	0	2	8	9	2	0

Chi-square test showed that there was no significant difference in problem difficulty among three experimental conditions: χ_(6)_^2^ = 5.507, *p* = 0.493, which indicating that different experimental condition did not affect the solution of the problem. There was also no significant difference in the sense of surprise: χ_(8)_^2^ = 5.548, *p* = 0.698. In addition, most of the participants thought that free observation phase was the most helpful to solve the problem (*M* ±*SD* = 71.78 ± 3.22).

#### Response Accuracy and Saccade Counts Analyses

To test whether the prototype heuristic effect exists in the attention tracing guidance by embodied attention, we analyzed the response accuracy and saccade counts. If the effect exists, the response accuracy and the saccade counts in prototypical condition would be higher than other conditions. Otherwise, there was no difference. The specific results of response accuracy and saccade counts were shown in Table 3.

**Table 3:** The response accuracy and saccade counts under different experimental conditions.

		Saccade counts (s/one)		
Guidance condition	Experiment samples	Free observation stage	Digital tracking task	Response accuracy
Prototypical	20	2.43 ± 0.38	1.51 ± 0.38	11 (55%)
Non-prototypical	19	2.13 ± 0.55	0.81 ± 0.29	5 (26.3%)
Non-guidance	21	2.50 ± 0.62		4 (19%)

The Chi-square test showed that there was a significant difference in response accuracy: χ_(2)_^2^ = 6.575, *p* = 0.037. There was a marginal significant difference in the response accuracy in the prototypical guidance and the non-prototypical guidance condition: χ_(1)_^2^ = 3.313, *p* = 0.069. There was a significant difference in the response accuracy in the prototypical guidance and the non-guidance condition, χ_(1)_^2^ = 4.262, *p* = 0.039. There was no significant difference in the response accuracy in the prototypical guidance and non-prototypical guidance condition, χ_(1)_^2^ = 0.302, *p* = 0.583. It indicated that the prototypical cue had heuristic effect on the insight problem solving.

An one-way ANOVA on saccade counts in free observation phase showed that there was no significant difference under different guidance condition: *F*_(2,57)_ = 2.579, *p* = 0.085, $\eta_{p}^{2}$= 0.067, indicating that participants in three conditions performed similar saccade counts in free observation phase. In other world, digital tracking task did not affect participants’ saccade counts in the free observation phase. We analyzed saccade counts in digital tracking task and showed that there was a significant difference in the prototypical guidance and the non-prototypical guidance condition: *t*_(29)_ = 5.577, *p* < 0.001, *d* = 1.03. And saccade counts in the prototypical guidance were significantly more than that in non-prototypical guidance condition. It suggested that the free observation phase and the digital tracking task were independent of each other, and the difference in eye movement in different guidance conditions was caused by the digital tracking task. It also indicated that the eye movement affected spatial cognitive activity implicitly.

### Discussion

The results of this experiment showed that most participants did not realize the connection between digital tracking task and problem solving, considering tracking task interfered with their thinking. It indicated that the connection between eye movement and spatial cognitive activity is implicit. The guidance of eye movement can affect their performance in solving insight problems. Response accuracy in prototypical guidance condition was significantly higher than non-guidance condition. And the non-prototypical guidance was not different significantly with prototypal guidance condition. The present experiment not only re-verify the effect of prototypical hint in insight problem solving heuristic effect, supporting (Grant and Spivey, 2003), viewing that the special mode of eye movements may play an embodied mechanism function. The results also enriched the theoretical background of embodied cognition: the process of cognitive activities can not only be offloaded in the environment, but also can produce interaction between our body and the surrounding environment, being influenced by the interaction in return. Comparing the results in the free observation phase and the digital tracking task phase, we can see that the differences in eye movement in different guidance conditions were caused by the digital tracking task. It not only suggested the free observation phase and the digital tracking task were independent of each other, but also indicated that the eye movement affected spatial cognitive activity implicitly. If participants realized the connection between digital tracking task and problem solving, those eye-movement trajectories should be similar during the free observation phase and the tracking task phase.

## Experiment 2B

### Materials and Methods

#### Participants

Sixty-two undergraduate students (38 female, mean age = 22.34 years, *SD* = 3.85) were recruited for course credit or proper reward. All participants reported normal or corrected-to-normal vision. They signed the informed consent and they had not participated in similar experiments before.

#### Design

The design of Experiment 2b was a single factor between-participants design. The independent variable is attention guidance type (attention-tracing, attention-transfer, and attention-fixation), and the accuracy, reaction time, and the saccade counts were dependent variable.

#### Materials

**FIGURE 10:**
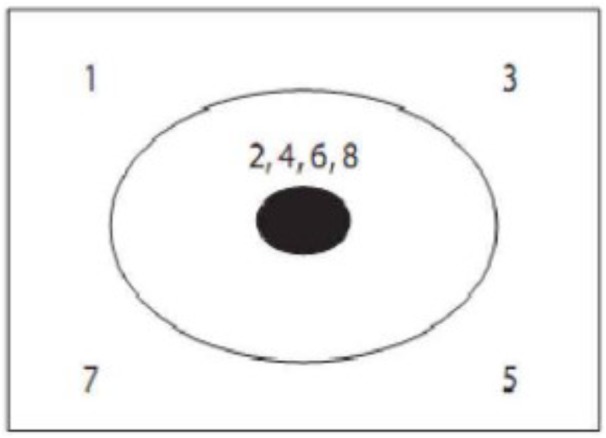
The digital tracking task of attention-tracing and attention-transfer condition.

**FIGURE 11:**
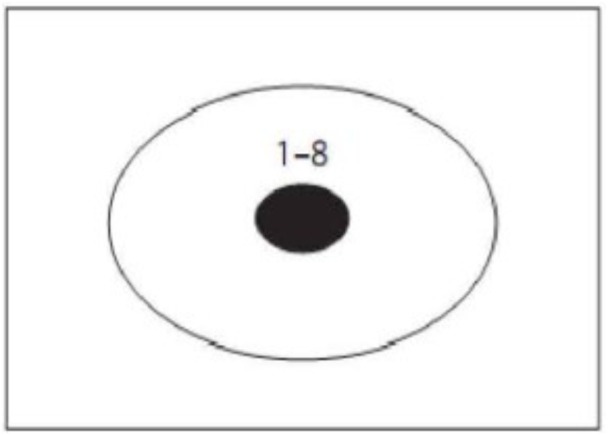
The digital tracking task of attention-fixation condition.

The materials were identical to Experiment 2a. The different types of attention guidance are operated by digital tracking task, and the digital tracking task in attention-tracing condition was shown in Figure 10 with different appearing position of digit: upper, middle, upper, middle, lower, middle, bottom left, middle; the participants in attention-tracing condition required were asked to fix on the stimulus (fixation should always attach to where the stimulus appears), and report the digit once found it. The digital tracking task in attention transfer condition was similar to that in attention tracing condition, as shown in Figure 10, with the difference that the fixation cannot follow stimulus moving and should always be fixed on the center of the screen only reported the digit once found it. The digital tracking task in attention-fixation condition was shown in Figure 11, requiring participants’ fixation follow stimulus. All stimuli appeared on the center of the screen.

#### Procedure

The apparatus was identical to Experiment 1. Sixty-two participants were randomly assigned to the experimental condition (attention-tracing condition, attention-transfer, and attention-fixation condition). The procedure was identical to Experiment 2a with the difference namely digital tracking task.

### Results

To test whether embodied effect of attention guidance on the problem solving need physical behavior, we analyzed the accuracy and response time in the digital task, even including the accuracy in problem solving, saccade counts in the free observation phase, and digital tracking task. Three participants realized the connection between guidance and problem solving, and other three participants whose accuracy didn’t reach 75% in digital tracing task, so these six data were excluded in final analysis. Fifty-six valid data were analyzed.

#### The Digital Tracking Task and Response Accuracy Analyses

There was a significant difference in accuracy under different experimental conditions, *F*_(2,53)_ = 8.60, *p* = 0.001, $\eta_{p}^{2}$ = 0.347. The accuracy of digital tracking task in attention-fixation condition was significantly higher than that in attention-tracking and attention-transfer condition (*p* = 0.011, *p* = 0.001). There was no significant difference between attention-tracking and attention-transfer condition (*p* = 0.525). The results of the reaction time in digital tracking task showed that there was a significant difference under different experimental conditions, *F*_(2,53)_ = 4.599, *p* = 0.014, $\eta_{p}^{2}$ = 0.159. Participants took shorter time in attention-fixation than attention-transfer condition (*p* = 0.012). There were no other significant differences (*p* > 0.05).

The Chi-square test showed that there was a significant difference in response accuracy under different experimental conditions, χ_(2)_^2^ = 8.875, *p* = 0.012. There was no difference in attention-tracking and attention-transfer condition (*p* > 0.05). The accuracy in attention-tracking and attention-transfer was significantly higher than that in attention-fixation condition (*p* = 0.007, *p* = 0.013).

#### The Saccade Counts Analyses

**FIGURE 12:**
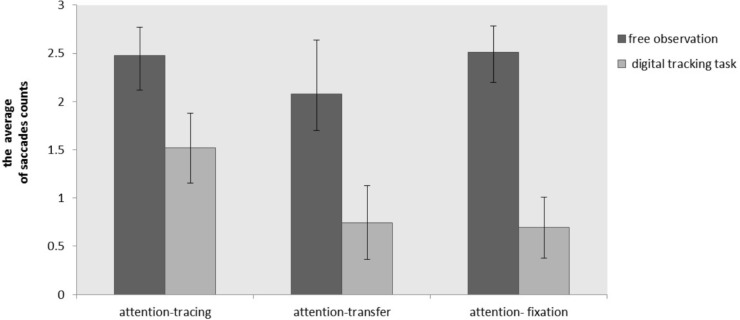
The average saccade counts of two phases under different guidance conditions (error bars: 95% confidence interval).

The average saccade counts in free observation phase and digital tracking task under different attention guidance were shown in Figure 12.

An one-way ANOVA saccade counts showed that there was a significant difference in saccade counts during the free observation phase under different attention guidance conditions, *F*_(2,53)_ = 3.748, *p* = 0.030, $\eta_{p}^{2}$ = 0.141. Saccade counts in attention-fixation were significantly higher than that in attention-transfer condition (*p* = 0.036). There were no other significant differences (*p* > 0.05). For different attention guidance conditions, there were significant differences in saccade counts in the digital tracking task, *F*_(2,53)_ = 28.370, *p* < 0.001, $\eta_{p}^{2}$ = 0.521. Attention-tracking and attention-transfer condition were significantly more than attention-fixation condition (*p* < 0.001), there was no significant difference in attention-tracking condition and attention-transfer condition (*p* = 0.643).

The above results indicated that the embodied effect of attention guidance on the problem solving did not need physical activity necessarily. Even though there was no physical activity involved, the attention transfer can also promote the emergence of insight.

### Discussion

The results of Experiment 2b showed that the difference of accuracy between attention-tracking and attention-transfer condition was not significant. Compared with the attention-fixation condition, attention-tracking and attention-transfer condition enjoyed higher response accuracy. However, in free observation phase, saccade counts in attention-fixation condition were significantly more than that in the attention-transfer condition. So we can infer that spontaneously produced saccade through skin didn’t cause the increase of accuracy. The increase of accuracy was due to the eye movement of participants or the transfer of attention in a short time which inspired insight in a particular pattern. In other words, the embodied effect of attention guidance on the problem solving not necessarily physical behavior involved.

The results showed that the accuracy in attention-fixation condition was higher than that in the attention-tracking condition and attention-transfer condition, and reaction time in the attention-fixation condition was shorter than that in the attention-tracking condition and attention-transfer condition. This is understandable because the fixation of participants kept fixing in the center of the screen in attention-fixation condition, and the stimulus for tracking also appeared in the center. However, there was no significant difference of accuracy and response time in the digital tracking task between attention-tracking and attention-transfer condition. It interpreted that the saccade counts in attention-tracking condition and attention-transfer condition may enjoy the same cognitive mechanism. And the increase of accuracy in attention-tracing condition may not be caused by the eye movement itself but by the attention transfer prior to the eye movements.

## General Discussion

The current study had four main important findings. First, the results of Experiment 1 showed that embodied gesture promoted the insight problem solving, and participant focused more on the number in formula in both two kinds of problems. According to the representational theory, the problem of matchstick can only be solved by transforming its representation to break through the dilemma and insight occurs (Knoblich et al., 1999). Participants focused on the number more than the symbol in the symbol constraint problems, which easily led participants to represent improper problem representations and then prevent the process of the problem solution. Thus, that’s why the accuracy was lower than that in numerical constraint problem.

Some researchers claimed that the influence of gesture in problem solving may result from space compatibility of the visual space template in working memory. Tversky (2004) found that gesture could unload and organize space working memory to improve problem solving. Previous studies have confirmed that hand gestures could maintain the outcome of study (Cook et al., 2008), which results from that gesture provides a representation which needed comparatively less cognitive resource, and then sparse cognitive resource could be used to record new information. Other studies have verified that working memory for using both language and hand gesture to express information is less than that for using language only, so participants can use strategy occupied less working memory to represent and learn (Goldin-Meadow, 2001; Wagner et al., 2004). Wagner et al. (2004) proved that the gesture itself boosted the encoding of long-term memory in the research. Grant and Spivey (2003) thought that indication of gestures could lead participants to correspond the mental representation of the problem and the related position and spatial information of the problem. And which is good for gesture perception activation in mind and for representation of space-related position. Hung et al. (2018) found that video lectures could improve people’s understanding and retention of knowledge. Thus, even if participants did not aware these representation activation, the gesture was also good for them to put the movement of matchsticks in their spatial representation (Penz and Ghosh, 2016). It may be interpreted that the participants were guided to combine their own gestures to solve the same type of problems, and the performance would be better than that under verbal instructions.

Second, the result of Experiment 2a showed that the guidance of eye movement affected their performance in solving insight problems and prototypical guidance cuing facilitated participants to solve the problem. Attention guidance had prototype heuristic effect in insight problem solving. The result could be interpreted by the prototype heuristic theory. The theory indicated that the process of insight problem solving is a process of prototype heuristic. In this process, if the proper prototype and its key heuristic information in the mind could be activated, individuals could break through the dilemma and solve the problem (Zhang et al., 2004). The key heuristic information refers to the information that plays a key role in solving the problem, and the activation of key heuristic information is a controllable and explicit process (Cao et al., 2006; Zhen-Zhen, 2008). Thus, compared with non-prototypical guidance cuing and no cuing, prototypical guidance cuing comprised more heuristic information, which was benefit for activating the prototype of the solution and its key heuristics information and then facilitated the insight problem solving. However, compared with prototypical guidance cuing, non-prototypical guidance cuing similarly guide participants to pay attention to the key area of problem solving, but didn’t significantly improve the performance of problem solving, which may resulted in non-prototypical guidance didn’t provide participants with key heuristics information in problem solving. Although a study have proved that when attention was guided to the key area of problem, the performance would be facilitated (Groen and Noyes, 2010), but if the key area didn’t include the key information to solve problem, participants could not reach for insight. It demonstrated that activating key heuristic information was important for insight problem solving.

Third, the result of Experiment 2b showed that attention tracing and attention transferring in prototypical guidance condition both facilitated the solution of insight problem. Thus, we concluded that saccades in attention-tracing condition may have the similar mechanism with that in attention-transfer condition. In the process of visual–spatial motion, there is close relation between attention and saccades. Saccades refer to physical performance of visual information extracting, which reflect the selection pattern of individual processing visual information, and it has direct or indirect relation with consciousness. Saccades are one fundamental representation of eye movement, which is quick moving of fixation. Godijn and Pratt (2002) found that the position of attention transfer is the same as the saccades (Godijn and Pratt, 2002). And some of the studies that followed supported this result (Peterson et al., 2004; Song and Hao, 2010; Kristjánsson, 2011). However, some researches showed that attention is not consistent with eye movement (Lawrence et al., 2004; Belopolsky and Theeuwes, 2009). At present, there are some hypothesis explaining the relation of saccades and attention. One was independent hypothesis, which demonstrated that one system could not control attention and saccades at the same time so they are separated. Another was reciprocal relation hypothesis, which demonstrated that the two processes of attention and saccades share some resource in the procedure of cognitive motion so the interaction exists. The preparation to move to some position could enhance the distribution of attention on the circular position and when attention was attached to objective position the incubation period of saccades would be shorter. And another was functional relation hypothesis, which believed that the relation between attention and saccades depended on how to explain the importance of circular events, if the circular event is not important, participants would not transfer their attention (Song and Hao, 2010; Song and Wang, 2012). Thus, the data of the present study supported the reciprocal relation hypothesis, because we found there was no difference between attention tracing and attention transfer. Song and Wang (2012) also found that saccades and attention transfer shared some resource in certain cognitive period, and saccades inferring certain position could facilitate the distribution of attention to circular positions. Meanwhile, saccades and attention transfer were not equal completely, because we can transfer our attention while we keep focusing, but can no keep attention while moving our eyes in the same time.

The final point is that the process of insight problem solving is unconsciously implicit activating process. At present, there are three arguments concerning insight problem solving: one is whether the mechanism underlying insight problem solving is consciously explicit searching process or unconsciously implicit activating process. Our data supported that insight problem solving is unconsciously and implicitly activating process and participants unconsciously process the relation of guidance and insight problem. Growing evidence has suggested that the process of insight problem solving is largely governed by an implicit learning mechanism that detects the differences between current and goal states, and regulates the strengths of the operators (Suzuki and Fukuda, 2013; Suzuki et al., 2014; Ball and Litchfield, 2017; Lebed and Korovkin, 2017). Suzuki and Fukuda (2013) study found that unconscious nature of insight problem solving was operator modulating the strengths during the impasse gradually, and roles of subliminal hint information in the problem solving processes. Consequently, the participants subconsciously used the gesture as a cue to facilitate insight problem solving. Gao and Zhang (2014) suggested that creative problem solving can be modulated by unconscious processing of enlightening information. Therefore, although participants didn’t realize the indicated relation of digit tracking task and to-be-solved problem, participants who moved eye fixations and transferred attention performed better.

Overall, this research mainly investigated the process of prompting insight problem solving and the nature of the embodied effect of insight problem solving. The findings of this experiment proved that embodied gesture and attention can promote the problem solving, and the result supported the reciprocal relation hypothesis between attention and saccades. It is important to note that we did not directly explore the brain mechanism of embodied action facilitating insight problem solving, but previous studies found that the temporal lobe played an important role in insight problem solving (Kounios and Beeman, 2013; Shen et al., 2017). And there were mainly four insight-activated brain regions, including the right medial frontal gyrus, the left inferior frontal gyrus, the left amygdala, and the right hippocampus. Importantly, various brain regions were variably activated during the four stages, and the gesture might lead to activation of one brain region and then help improved performance Shen et al. (2018a). However, the exact activated brain region was still not clear in the process of embodied action facilitating insight problem solving, and it should be studied further.

## Conclusion

Embodied gesture could facilitate the performance of insight problem solving, which indicated that embodied gesture enhance insight problem solving and gesture guidance was better than speech guidance. Compared with non-prototypical guidance cuing and no cuing, prototypical guidance cuing was the best cuing in insight problem solving. Attention guidance had prototype heuristic effect in insight problem solving. Attention tracing and attention transferring in prototypical guidance condition both facilitated the solution of insight problem, which supported the reciprocal relation hypothesis of saccades and attention. Embodied guidance facilitated insight problem solving implicitly.

## Ethics Statement

The study reported in the manuscript entitled “The Effect of the Embodied Guidance in the Insight Problem Solving: An Eye Movement Study” has been approved by the Institutional Review Board at Guangzhou University.

## Author Contributions

All authors listed have made a substantial, direct and intellectual contribution to the work, and approved it for publication.

## Conflict of Interest Statement

The authors declare that the research was conducted in the absence of any commercial or financial relationships that could be construed as a potential conflict of interest.
